# Acoustic characterization of low intensity focused ultrasound system through skull

**DOI:** 10.1186/2050-5736-3-S1-P13

**Published:** 2015-06-30

**Authors:** Meghedi Babakhanian, Bryan Nowroozi, George Saddik, Ashkan Maccabi, Neha Bajwa, Warren Grundfest

**Affiliations:** 1 University of California at Los Angeles, Los Angeles, California, United States

## Background/introduction

Low Intensity Focused Ultrasound (LIFU) continues to gain traction in the field of non-invasive neuromodulation. Recent *in vivo* rat studies suggest that LIFU can be used to modulate region-specific brain activity in the motor cortex, leading investigators to conclude that LIFU may be a better alternative to more prominent, and more invasive, mechanisms of neuromodulation for conditions such as Parkinson’s disease, Epilepsy, and applications such as drug delivery applications. Despite these recent successes, a comprehensive understanding of beam patterns within the skull remains elusive.

## Methods

LIFU neuromodulation requires precise targeting within the brain. The skull deforms the beam and makes the localization and precise energy deposition difficult to determine in both animal models and humans. We hypothesize that a better understanding of beam deformation will improve our ability to compensate for the effects of the skull, thereby enhancing targeting during focused ultrasound mediated neuromodulation.

## Results and conclusions

In preparation for *in vivo* LIFU efficacy studies, we examined beam deformation in rat skulls, 2.8mm inferior from the bregma. We obtained beam patterns using a precision acoustic measurement tank (AIMS, Sonora/Unisyn), Olympus focused V301-SU transducer, and a precision hydrophone, targeting different regions of the brain.

Specifically, we investigated intensity attenuation, beam shape modulation, focal accuracy, and reverberation within the skull. Preliminary data from ten rat skulls reveal that beam intensity, beam shape, and targeting are all significantly affected during transcranial sonication, and that changes in these parameters must be taken into account to accurately target specific neuronal structures. This suite of studies will enhance our understanding of beam targeting, accuracy and precession *in vivo*. In addition, insights gleaned from this approach are expected to promote new avenues of clinical applications for the treatment of drug delivery, gene therapy and neurological illnesses.

